# Short-Lived IFN-γ Effector Responses, but Long-Lived IL-10 Memory Responses, to Malaria in an Area of Low Malaria Endemicity

**DOI:** 10.1371/journal.ppat.1001281

**Published:** 2011-02-10

**Authors:** Jiraprapa Wipasa, Lucy Okell, Supachai Sakkhachornphop, Chaisuree Suphavilai, Kriangkrai Chawansuntati, Witaya Liewsaree, Julius C. R. Hafalla, Eleanor M. Riley

**Affiliations:** 1 Research Institute for Health Sciences, Chiang Mai University, Chiang Mai, Thailand; 2 Department of Immunology and Infection, Faculty of Infectious and Tropical Diseases, London School of Hygiene and Tropical Medicine, London, United Kingdom; 3 Vector Borne Disease Section, Office of Disease Prevention and Control, Chiang Mai, Thailand; Case Western Reserve University, United States of America

## Abstract

Immunity to malaria is widely believed to wane in the absence of reinfection, but direct evidence for the presence or absence of durable immunological memory to malaria is limited. Here, we analysed malaria-specific CD4^+^ T cell responses of individuals living in an area of low malaria transmission in northern Thailand, who had had a documented clinical attack of *P. falciparum* and/or *P. vivax* in the past 6 years. CD4^+^ T cell effector memory (CD45RO^+^) IFN-γ (24 hours *ex vivo* restimulation) and cultured IL-10 (6 day secretion into culture supernatant) responses to malaria schizont antigens were detected only in malaria-exposed subjects and were more prominent in subjects with long-lived antibodies or memory B cells specific to malaria antigens. The number of IFN-γ-producing effector memory T cells declined significantly over the 12 months of the study, and with time since last documented malaria infection, with an estimated half life of the response of 3.3 (95% CI 1.9–10.3) years. In sharp contrast, IL-10 responses were sustained for many years after last known malaria infection with no significant decline over at least 6 years. The observations have clear implications for understanding the immunoepidemiology of naturally acquired malaria infections and for malaria vaccine development.

## Author Summary

Despite some recent successes in reducing the burden of malaria in several African countries, malaria still causes up to 500 million cases of acute illness every year, killing over a million people. The widespread availability of a safe and effective vaccine would greatly increase our chances of controlling this disease and possibly, even, eliminating it as a major health concern. Attempts to develop a vaccine have had limited success. The fact that people can be repeatedly infected with malaria over many years has raised the concern that immunity to malaria may be short-lived, complicating the induction of long term protection by vaccination. In this study we have calculated the half-life of cellular immune responses to malaria in previously infected individuals from Thailand. We have found that, in the absence of boosting of immunity by reinfection, malaria-specific inflammatory responses are relatively short-lived, with a half life of approximately 3 years. However, malaria-specific anti-inflammatory responses (which have been linked to resistance to severe malarial disease) seem to be very long-lived (the half life being indistinguishable from infinity). Our observations have important implications for understanding the immunoepidemiology of naturally acquired malaria infections and for malaria vaccine development.

## Introduction

It is well established that immunity to severe clinical symptoms of malaria is acquired rapidly, but immunity to malaria infection is slow to develop and incomplete [1], [2]. Naturally acquired protective immunity against blood stage malaria involves both antibodies and CD4^+^ T cells (reviewed in [2]). Antibodies provide protection by blocking invasion of merozoites into new red blood cells (RBCs), blocking cytoadherence of infected RBCs (iRBCs) to endothelial cells, and enhancing phagocytic activity of monocytes and macrophages. CD4^+^ T cells play crucial roles by providing help to B cells for the production of antibodies and by producing immune mediators essential for regulating cellular immune effector mechanisms.

Although the contribution of CD4^+^ T cells to blood-stage malaria immunity has been extensively studied, the development and maintenance of malaria-specific memory CD4^+^ T cells is not well understood. It has been proposed that antigenic diversity [3], inhibition of maturation of dendritic cells [4], [5], and apoptotic deletion of malaria-specific T cells [6], [7] impair the development of memory responses after malaria infection, in particular impeding the development and/or longevity of memory CD4^+^ T cells. However, studies in animal models of malaria infection indicate that memory CD4^+^ T cells do develop and are maintained normally after malaria infection [8], [9]. Whether the results from these experimental infections are representative of responses in humans remains to be elucidated.

Memory CD4^+^ T cells typically respond to lower doses of antigen, require less costimulation, and rapidly differentiate into cytokine-producing effector cells after encounter with specific antigen [10]. They are characterized by expression of surface markers such as CD62L (L-selectin), CD45RO and lack of CCR7 [11] but it is becoming clear that the pool of CD4^+^ memory T cells against any particular pathogen is phenotypically and functionally heterogeneous [12].

Understanding the development and maintenance of memory CD4^+^ T cells is fundamental to vaccine development. However, the presence of substantial numbers of malaria-reactive memory T cells in malaria naïve individuals [13], [14], [15] makes it difficult to interpret and understand the longevity of malaria-specific memory T cells. In the present study, we have identified malaria-specific cellular immune parameters among malaria-exposed individuals living in an area of very low malaria endemicity in Northern Thailand and determined the duration of the memory CD4^+^ T cell response to *P. falciparum* under conditions of infrequent re-exposure/boosting of the immune response.

## Materials and Methods

### Ethics statement

Fully informed, written consent was obtained from each participant prior to enrolment in the study. Ethical approvals were obtained from the research ethics committees of the Research Institute for Health Sciences at Chiang Mai University, of the Ministry of Public Health, Thailand and of the London School of Hygiene and Tropical Medicine, UK.

### Study area and subjects

Study subjects were recruited from among long-term adult residents of Muang Na, a village in the malaria endemic Chiang Dao region of northern Thailand, near the border with Myanmar, or were permanent adult residents of the city of Chiang Mai where malaria transmission does not occur [16].

Venous blood was collected in acid citrate dextrose on the day of recruitment and again 3, 6 and 12 months after recruitment. Giemsa-stained blood films were examined for the presence of malaria parasites. As HIV infection may affect immunological parameters, all subjects were tested for HIV infection (presence of anti-HIV antibodies by gel particle agglutination assay) at the time of recruitment and at the end of the study; subjects received pre- and post-test counseling from trained HIV counselors. Data from HIV-infected subjects were excluded from the analysis. None of the subjects were infected with *P. falciparum* or *P. vivax* - as determined by blood film examination and PCR - at any visit.

### Parasites

The laboratory 3D7 strain of *P. falciparum* was maintained in continuous culture under standard conditions, as described previously [17]. Cultures were periodically tested for mycoplasma contamination by polymerase chain reaction (PCR) (Venor GeM, Minerva Biolabs) and found to be mycoplasma free. Mature schizonts were obtained by gradient centrifugation over 60% Percoll (Amersham Biosciences), adjusted to a concentration 1×10^8^ schizont-infected red cells/ml and exposed to three freeze/thaw cycles to obtain *P. falciparum* schizont extract (PfSE). A single batch of PfSE was used throughout the study. The initial batch was aliquoted and kept frozen at −80^o^C until required.

### Detection of malaria parasites by nested PCR

Blood samples from each subject were checked for subpatent malaria parasitaemia by PCR. DNA was isolated using FlexiGene DNA extraction kits (Qiagen) according to the manufacturer's protocol and subjected to nested PCR for *P. falciparum* and *P. vivax,* as described previously [18].

### Preparation of peripheral blood mononuclear cells (PBMC)

PBMC were separated from citrated blood by gradient centrifugation over Ficoll-Hypaque (Amersham Biosciences). Contaminating erythrocytes were removed by incubation with lysis buffer (0.15 M NH_4_Cl, 10 mM KHCO_3_, 0.1 mM Na_2_EDTA) at RT for 5 minutes. The cells were washed twice with RPMI, resuspended in 10% human AB serum/RPMI (R10) culture medium, stained with trypan blue to identify viable cells, counted and adjusted to the required concentration.

### Immunophenotyping

Freshly isolated PBMC were phenotyped by staining 5×10^5^ PBMCs with fluorochrome-conjugated anti-human CD3, anti-human CD4 and anti-human CD45RO (all from Caltag) for 30 min at 4°C. Cells were washed twice with PBS containing 1% foetal bovine serum (FBS, Gibco) and 0.05% sodium azide (FACS buffer), fixed in 1% paraformaldehyde (PFA) in PBS, and analysed by flow cytometry (FACSCalibur, Becton Dickinson) using Cell Quest software. Cell integrity was ascertained from their FSC/SSC distribution.

### 5-(and –6)-carboxyfluorescein diacetate succinimidyl ester (CFSE) labeling

PBMC were washed twice with sterile PBS and adjusted to 1×10^7^ cells per ml in 0.1% FBS in PBS. The cells were then stained with CFSE (Molecular Probes) at a final concentration of 10 µM at RT for 8 min. Labeled cells were washed three times with 5% FBS in PBS, adjusted to 1×10^6^ cells/ml in R10 and stimulated with 10 µg/ml PPD, 5 µg/ml PHA, 5×10^5^/ml schizont equivalents of PfSE or medium alone for 6 days at 37°C, 5% CO_2_. Labeled cells were then stained with mAbs as described above.

### Intracellular cytokine staining

Two million PBMC were stimulated with 10 µg/ml PPD, 5 µg/ml PHA, 5×10^5^/ml schizont equivalents of PfSE, or medium alone at 37°C, 5% CO_2_ for 20 hours. Brefeldin A (Sigma) at a final concentration of 10 µg/ml was added to cultures for the last 4 hrs of incubation. Each culture was divided into two aliquots, washed with FACS buffer and stained with anti-human CD3-Tri-colour, anti-human CD4-APC and anti-human CD45RO-FITC for 30 min at 4°C. The cells were fixed with 4% PFA, permeabilized with 0.2% saponin (Fluka) in FACS buffer for 10 min at RT and incubated with either anti-human IFN-γ-PE or anti-human IL-10-PE for 30 min at 4°C, washed and then fixed in 1% PFA. Appropriate isotype controls were used. The cells were analysed by flow cytometry, in which 30,000 lymphocytes were collected.

### Measurement of IL-10 production by ELISA

Two million PBMC were stimulated with 10 µg/ml PPD, 5 µg/ml PHA, 5×10^5^/ml schizont equivalents of PfSE, or medium alone at 37°C, 5% CO_2_ for 6 days. Culture supernatants were analysed for IL-10 by ELISA. Maxisorb immunoplates (Nalge Nunc International) were coated with 2 µg/ml of purified anti-human IL-10 (BioLegend) in coating buffer overnight at 4°C. Plates were washed, blocked with 1% BSA/PBS for 2 hr at RT and washed. One hundred microlitres of supernatants or serially diluted standard cytokines in sample diluent (0.05% Tween-20 in blocking buffer) were added to each well, plates were incubated at RT for 2 h, washed and then incubated for 1 hr at RT with 100 µl of 1 µg/ml biotinylated anti-IL-10 (BioLegend). Plates were then developed with Streptavidin-HRP (R & D Systems) and OPD/H_2_O_2._ The colorimetric reaction was stopped with 2 N sulphuric acid and absorbance read at 492 nm on a Spectra MR plate reader (Dynex Technology). Sample values were calculated by interpolation from a standard curve of recombinant IL-10 which was included on every plate. Standard curves were compared (between and within batches) and plates with substantial deviations were repeated. Samples were processed in date order with most plates containing samples from different groups and/or from different follow-up visits.

### Statistical analysis

Statistical analysis was performed as described previously [16]. Briefly, Mann Whitney U test was used to analyse differences in the T cell responses among groups (GraphPad Prism Software). Decay rates of memory CD4 T cell responses were calculated using logarithmically transformed data. The effect of time since last malaria infection was analysed using a log-linear mixed-effects regression model incorporating random intercepts assuming a Gaussian distribution across individuals. This resulted in an estimate of the decay rate of memory T cell responses, assuming a single-exponential decay model. Half-lives were calculated from the estimated decay rate and the boundaries at 95% confidence interval obtained from the mixed-effects model. Where the decay rate is a positive value, the calculated half-life is reported as infinity. All analyses were undertaken using Stata (version 10, Statacorp LP).

## Results

### Characteristics of study subjects at recruitment

Three groups of study subjects were recruited based on their place of residence and their prior malaria history. Of the 93 subjects originally recruited [16], sufficient PBMC from 87 subjects were available for this part of the study. The characteristics of these 87 subjects are summarized in Table 1. Subjects from Chiang Mai were designated “City Naïve” (n = 17). Subjects from Muang Na (Chiang Dao) were designated “Rural with no clinical malaria episode” (Rural 1; n = 29) if they reported no prior episodes of malaria infection and/or if no record of malaria infection was found in the past 6 years. Muang Na residents who had one or more fully documented episodes of infection with *P. falciparum, P. vivax* or both parasite species were designated as “previously malaria infected” (Rural 2; n = 41). Among these 41 subjects, 21 (51.2%) individuals were known to have had at least one episode of *P. falciparum* infection, 14 (31.1%) were known to have had at least one episode of *P. vivax* infection and 6 (14.6%) individuals were known to have been infected with both *P. falciparum* and *P. vivax* in the past 6 years. None of the subjects were positive for *P. falciparum* or *P. vivax* as determined by blood film examination and PCR at any study visit. The three groups did not differ significantly by age or sex.

**Table 1:** Characteristics of study subjects at recruitment.

	City	Rural 1	Rural 2
Number of subjects recruited	17	29	41
Male N (%)	8 (47.1)	9 (31.0)	22 (53.7)
Age (years) Mean ± SD	34.7±8.2	32.5±8.7	34.0±7.4
Age Range (years)	23–46	19–49	19–48
Individuals with recorded malaria episodes^a^			
* P. falciparum* only N (%)			21 (51.2)
* P. vivax* only N (%)			14 (31.1)
* P. falciparum* and *P. vivax* N (%)			6 (14.6)
Number of malaria episodes per person a,b			
* P. falciparum* Mean ± SD (Range)			1.3±0.6, 1–3
* P. vivax* Mean ± SD (Range)			1.1±0.3 (1–2)
Time since last malaria episode (months)^b^			
* P. falciparum* Mean ± SD (Range)			21.2±12.9 (4–58)
* P. vivax* Mean ± SD (Range)			20.6±10.1 (7–39)

a Data extracted from records of the Office of Vector Borne Disease Control, Department of Communicable Diseases Control, Ministry of Public Health, Thailand.

b Malaria episodes that occurred within 45 days were considered as a single episode.

### Proliferating CD4^+^ T cells exhibit an effector memory phenotype

**Figure 1:**
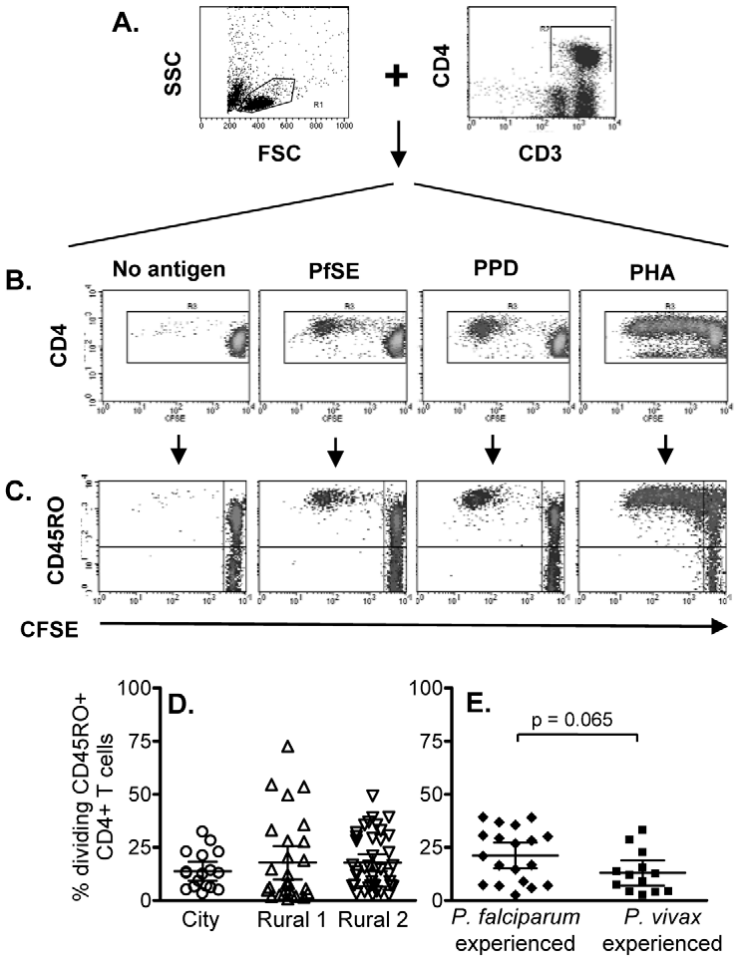
Flow cytometric characterization, frequencies and longevity of memory CD4 T cells proliferating in response to PfSE. CFSE-labeled PBMCs were stimulated with no antigen, PfSE, PPD or PHA for 6 days. Cells were harvested and stained with fluorochrome-conjugated anti-CD3, CD4 or CD45RO mAbs. (A) PBMC were gated by forward and side scatter and the CD3^+^ and CD4^+^ T lymphocyte population was identified; (B) CFSE expression in CD4^+^ T cells. CD3^+^ CD4^+^ T cells were gated and CFSE expression examined in relation to CD45RO (C) expression. The plots demonstrate the results for one representative subject known to have been infected previously by *P. falciparum*. Percentages of proliferating CD45RO^+^ CD4^+^ T cells (CFSE^low^) among (D) city (circles), Rural 1 (triangles) and Rural 2 (reverse triangles) subjects and (E) between individuals who had been infected with *P. falciparum* and *P. vivax*. Mean ±95% confidence interval is shown for each group.

It has previously been shown that T cells from naive (malaria non-exposed) volunteers proliferate in response to *Pf* schizont extract (PfSE) and that these responses are likely due to recognition of cross-reactive antigens [13], [15], [19]. The initial objective of this study was thus to determine whether there were differences in the proliferative capacity and the phenotype of proliferating cells amongst city (naïve) and rural (definitely and putatively exposed) individuals. Proliferation was assessed by CFSE dilution in CFSE-labeled PBMCs cultured with PfSE and subsequent phenotyping by flow cytometry. Since Thai people are routinely vaccinated with BCG (*Mycobacterium bovis* Bacille Calmette-Guérin), PPD (purified protein derivative of *Mycobacterium spp.)* was used as a positive control for a recall antigen response. PHA was used a control for cell viability. The flow cytometric gating strategy is shown in Figure 1 for a subject with known prior exposure to *P. falciparum*. CD3^+^CD4^+^ lymphocytes were gated (Figure 1A) and then analysed for CFSE levels (Figure 1B). CFSE dilution was also analysed separately for CD45RO^+^ and CD45RO^-^ cells (Figure 1C).

Significant cell division (i.e. accumulation of CFSE^low^ cells) was seen among CD4^+^ cells cultured with PfSE, PPD and PHA. In all cases, the majority of dividing cells were CD45RO^+^ suggesting that the responding cells are of the effector memory phenotype and confirming previous observations [20]. Consistent with published data, T cells from ‘City Naïve’ subjects proliferated in response to PfSE and their proliferative capacity did not differ from that of T cells from ‘Rural 1’ and from ‘Rural 2’ donors (Figure 1D; Supplementa1 Table S1). Similarly, there was no difference in the percentages of dividing cells (Figure 1E) between subjects who had previously been infected with *P. falciparum* only or with *P. vivax* only.

There were no consistent differences in PHA-induced or PPD-specific proliferative responses between City and Rural subjects although PHA responses were somewhat higher in Rural 1 subjects (Rural 1 vs. City naïve, p = 0.03) and PPD responses were higher in Rural 2 subjects (Rural 2 vs. City naïve, p = 0.004) (Supplemental Table S1).

### IFN-γ production by malaria-specific effector memory CD4^+^ T cells

**Figure 2:**
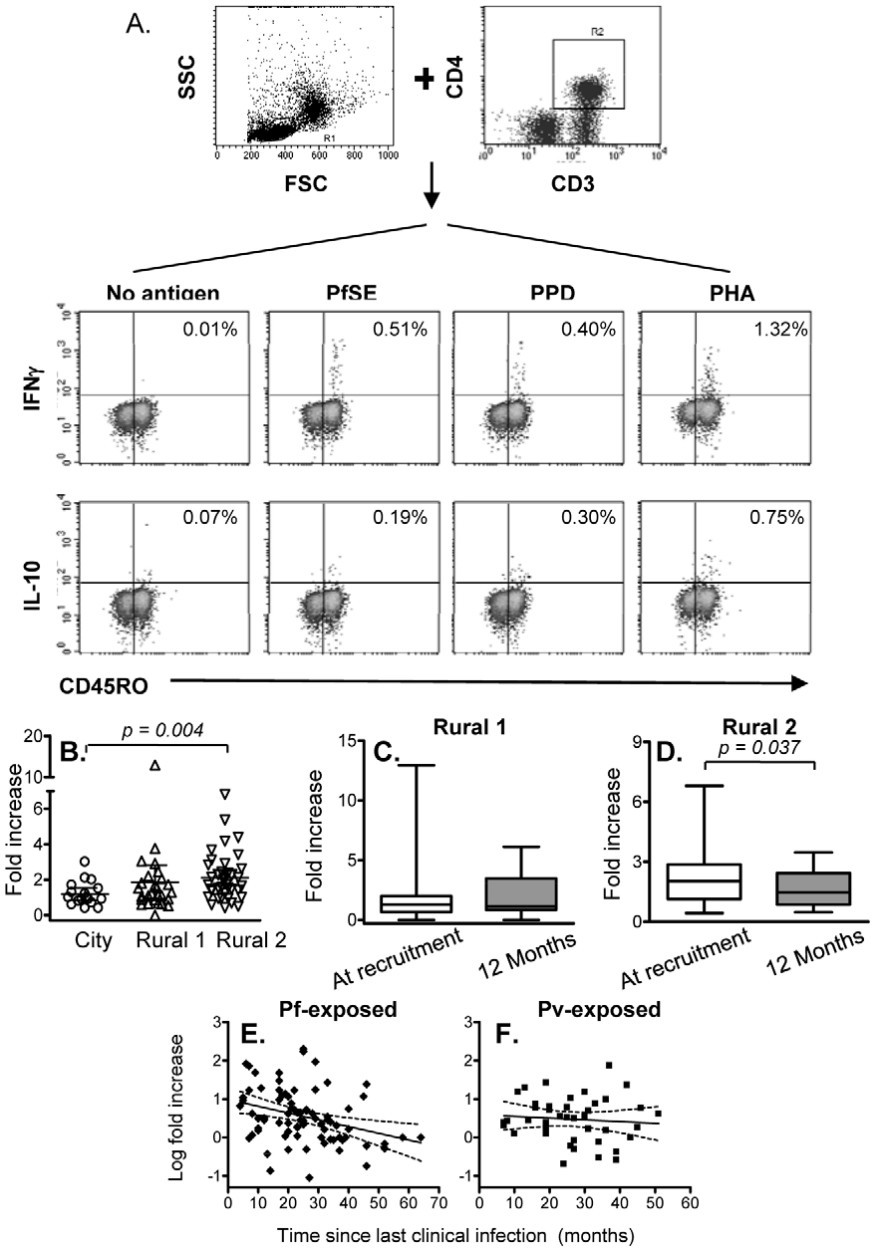
Immediate IFN-γ and IL-10 production by effector memory CD4 T cells. PBMCs were stimulated with no antigen, PfSE, PPD or PHA for 20 hours. Cells were harvested and stained with fluorochrome-conjugated anti-CD3, CD4, CD45RO, IFN-γ or IL-10 mAbs. (A) PBMC were gated by forward and side scatter and the CD3^+^ and CD4^+^ T lymphocyte population was identified. IFN-γ and IL-10 production in CD45RO^+^ populations were then determined. The plots demonstrate the results for one representative subject known to have been infected previously by *P. falciparum*. (B) Fold increase (mean ±95% CI) in IFN-γ production in response to PfSE (compared with cells cultured without antigen) at recruitment for each subject. Immediate IFN-γ response at recruitment compared to at 12 months later in Rural 1 (C) and Rural 2 (D) subjects. [Horizontal line  =  median; box  = 25^th^ and 75^th^ percentiles; whiskers  =  minimum and maximum values]. Paired *t*-tests were used to analyse differences between the two time points. Best-fit regression analysis of changes in fold increase of immediate IFN-γ producing CD45RO^+^ CD4^+^ T cells in response to PfSE over time in *P. falciparum*-exposed (E) and *P. vivax*-exposed (F) subjects. Solid lines represent best fit regression lines estimating the rates of decline of immediate cytokine responses over time and dashed lines represent the 95% CI. The median percentages of immediate IFN-γ producing CD45RO^+^ CD4^+^ T cells in unstimulated control cultures were 0.029, 0.031 and 0.027 in naïve, Rural 1 and Rural 2 subjects, respectively.

Since immunological memory depends on being able to mount a fast and effective response to infection, the number and/or function of effector memory cells is likely to be a more relevant indicator of an anamnestic response than simply the number or proportion of proliferating cells. We therefore examined the capacity of memory CD4^+^ T cells to produce the effector cytokine IFN-γ and the regulatory cytokine IL-10 in response to 24 hrs stimulation with PfSE or PPD. Representative flow cytometry plots showing intracellular IFN-γ and IL-10 among CD4^+^ CD45RO^+^ T cells are shown in Figure 2A. The number of IFN-γ^+^ or IL-10^+^ T cells in PfSE-stimulated cultures is shown as the fold increase compared with the number in unstimulated cultures.

Very few CD45RO^+^ CD4^+^ T cells from City Naïve or Rural 1 subjects produced IFN-γ when stimulated with PfSE (averaging 1.2 and 1.8 fold increase over background, respectively) (Figure 2B). By contrast, IFN-γ production in response to PfSE among memory CD4^+^ T cells of Rural 2 individuals (averaging 2.2 fold increase over background) was significantly higher than for City Naïve subjects (p = 0.004; Mann Whitney U test). Data from subjects who had been infected with *P. falciparum* only and *P. vivax* only were pooled since there was no difference in IFN-γ production between the two groups (data not shown), however exclusion of *P. vivax* only subjects did increase the level of significance of the difference between naïve and exposed groups (p = 0.002).

Median levels of PfSE-induced IFN-γ production among CD45RO^+^ CD4^+^ T cells did not change over the 12 months of the study in the Rural 1 group (Figure 2C) whilst in the Rural 2 group, median levels of PfSE-induced IFN-γ declined slightly but significantly (p = 0.037; paired *t*-test) over a period of 12 months (Figure 2D). Among the 41 rural subjects (Rural 1 + Rural 2) who had PBMCs tested at the beginning and at the end of the study, 23 (56.1%) responded to PfSE by making IFN-γ at the time of recruitment and all of these remained IFN-γ positive 12 months later. Six rural subjects (14.6%) who did not make IFN-γ at the beginning of the study did however make IFN-γ in response to PfSE at the end of the study, 12 months later. There was no indication that these 6 individuals were infected with malaria during the 12 months of the study and thus boosting of their cellular immune response is unlikely, although this cannot be ruled out. Alternatively, it is possible that in these individuals the frequency of circulating PfSE-responsive Th1 cells fluctuates close to the level of detection of the assay leading to stochastic variation in whether they are detected or not at any particular point in time.

PBMC from very few individuals produced IL-10 after short-term stimulation with PfSE and numbers of IL-10 positive cells were not significantly above control values (median fold increase 1.0, 0.9 and 1.2 for naïve, rural 1 and rural 2 individuals respectively). Thus, there were no significant differences in immediate IL-10 responses among the groups and no significant change in immediate IL-10 production in response to PfSE during the 12 months of study was observed (data not shown).

There were no differences between the groups in immediate IFN-γ responses to PPD and the magnitude of PPD-specific IFN-γ responses did not change over the 12 months of the study (Supplemental Figure S1).

### Longevity of IFN-γ responses among effector memory T cells in malaria-exposed subjects

To determine the longevity of the immediate memory T cell response to PfSE, we analysed the frequency of IFN-γ-producing memory cells versus time since last known infection, in Rural 2 individuals. Mixed-effects regression models indicate a steady decline in the IFN-γ response to PfSE in the *P. falciparum*-experienced individuals (Figure 2E). The best estimate for the half-life of PfSE-specific IFN-γ-producing CD4^+^ T cells in these individuals is 3.27 years (95% CI: 1.94–10.26 years) (Table 2). Although the decay of IFN-γ-producing T cells was much less obvious among *P. vivax-*experienced individuals (estimated half-life 12.6 years, although the 95% CI included infinity) (Figure 2F) it did not differ significantly from the half-life in *P. falciparum*-experienced individuals (p = 0.227).

**Table 2:** Longevity of memory CD4 T cell responses to PfSE in *P. falciparum*- and *P. vivax*-exposed subjects.

	No. of observations	Annual decline in log response (95% CI)	Half-life (95% CI)	p
*P. falciparum-*exposed subjects				
* *Immediate IFN-γ	78	−0.212 (−0.357, −0.068)	3.27 (1.94, 10.26)	**0.004**
* *Cultured IL-10	77	0.021 (−0.481, 0.523)	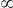 (1.44, 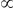 )	0.935
*P. vivax*-exposed subjects				
* *Immediate IFN-γ	41	−0.055 (−0.247, 0.137)	12.6 (2.81, 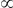 )	0.247
* *Cultured IL-10	38	0.294 (−0.601, 1.188)	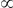 (1.15, 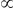 )	0.601

### IL-10 production by malaria-specific memory CD4+ T cells

**Figure 3:**
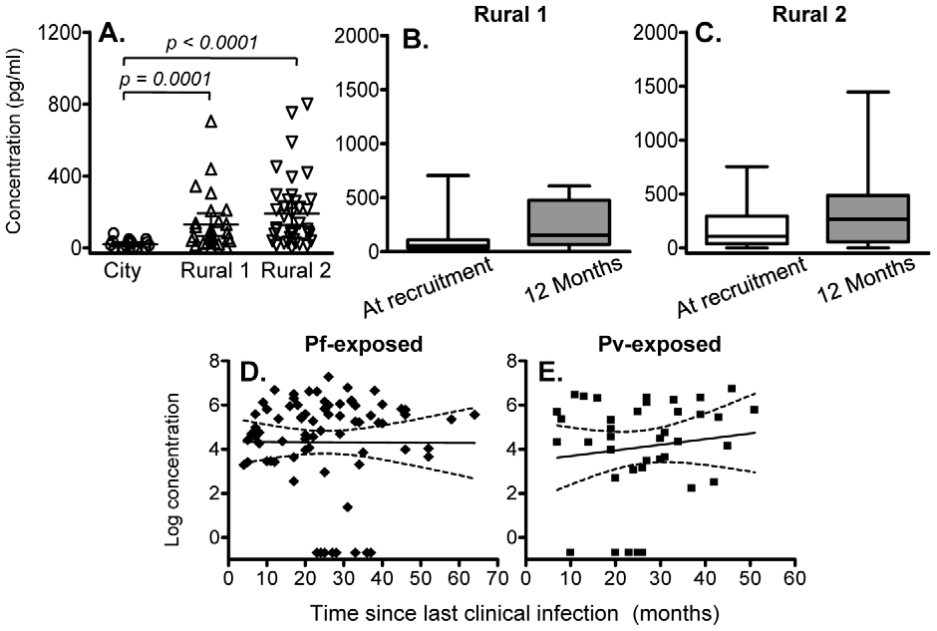
IL-10 concentrations in 6 day cell culture supernatants. (A) Concentration (mean ±95% CI) of IL-10 in 6 day, PfSE-stimulated culture supernatants at recruitment. IL-10 concentrations in 6 day, PfSE-stimulated culture supernatants at recruitment and 12 months later in Rural 1 (B) and Rural 2 (C) subjects. [Horizontal line  =  median; box  = 25^th^ and 75^th^ percentiles; whiskers  =  minimum and maximum values]. Paired *t*-tests were used to analyse differences between the two time points. Best-fit regression analysis of the 6 day IL-10 response to PfSE over time in *P. falciparum*-exposed (D) and *P. vivax*-exposed (E) subjects. Solid lines represent best fit regression lines estimating the rates of decline of the 6 day cytokine responses over time and dashed lines represent the 95% CI.

We found little evidence of malaria-specific IL-10 responses by flow cytometry after 24 hrs co-culture with PfSE, PHA or PPD. For PfSE, the fold increase in IL-10^+^ CD4^+^ T cells between PfSE-stimulated and unstimulated cells averaged 1.08 with no difference between groups and with very low median fluorescence intensity values (Figure 1A and data not shown). We therefore examined the accumulation of IL-10 in Day 6 cell culture supernatants as an indication of central memory responses [21]. Strikingly, although PfSE-stimulated PBMC cultures from Rural 1 and Rural 2 subjects contained similar concentrations of IL-10, in both cases IL-10 concentrations were significantly higher than in cultures of PBMC from City Naïve subjects (Figure 3A). Data from subjects who had been infected with *P. falciparum* only and *P. vivax* only were pooled since there were no differences in IL-10 production between the two groups and exclusion of *P. vivax*-exposed subjects did not affect our conclusions (data not shown). Interestingly, median concentrations of IL-10 were stably maintained in both Rural 1 and Rural 2 subjects over the 12 months of the study (Figure 3B, 3C) and mixed-effects regression models showed that PfSE-induced IL-10 concentrations were stably maintained for up to 6 years after last documented malaria infection in both *P. falciparum*- (Figure 3D) and *P. vivax*- (Figure 3E) exposed Rural 2 subjects. The best estimate of the half life of these IL-10 responses is infinity (Table 2).

Of note, there was no significant association between individuals making an IFN-γ response and those making an IL-10 response, i.e. individuals making IFN-γ were no more or less likely to make IL-10 than were individuals who did not make IFN-γ.

There were no significant differences between City and Rural subjects in Day 6 IL-10 responses to PPD (although responses were higher among Rural 2 than Rural 1 subjects) and the magnitude of PPD-specific IL-10 responses did not change over the 12 months of the study (Supplemental Figure S1).

### Immediate IFN-γ and 6 day IL-10 responses to malaria antigens are associated with humoral immunity to malaria

We have previously described long-lived antibody and memory B cell responses to malarial antigens in this same group of individuals [16]; only rural residents had detectable humoral immune responses to malaria antigens. To determine whether humoral and cellular immune memory to malaria are linked, we compared cellular immune parameters between seropositive and seronegative rural individuals and between those who did or did not have detectable memory B cell responses to malaria antigens.

**Figure 4:**
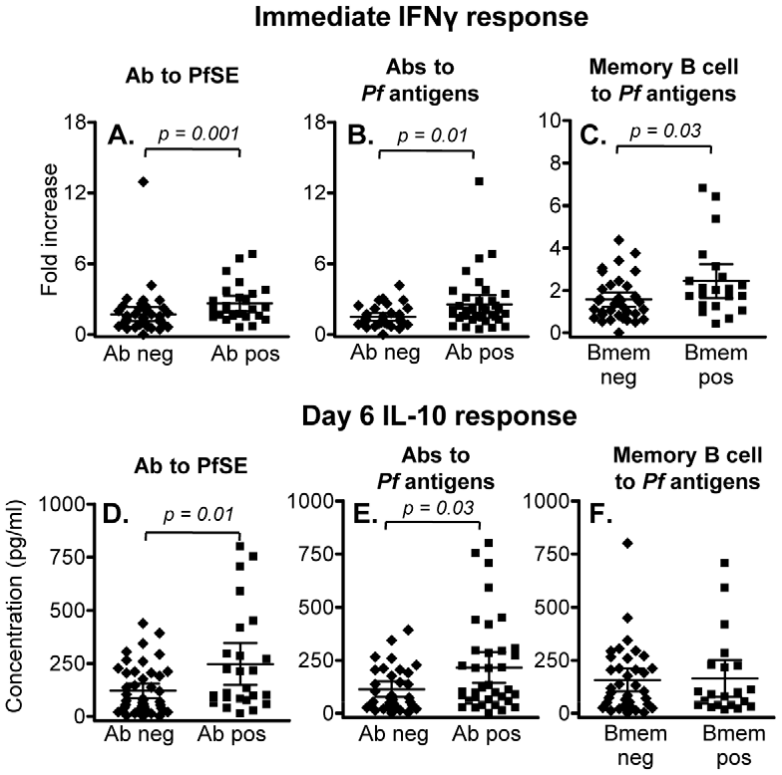
Association between memory T cell responses and humoral immunity to malaria. (A-C) Immediate IFN-γ responses in rural individuals with (Ab pos) or without (Ab neg) antibodies to (A) PfSE or (B) recombinant malaria antigens, or (C) individuals with or without detectable memory B cells specific for *P. falciparum* antigens. The median percentages of immediate IFN-γ producing CD45RO^+^ CD4^+^ T cells in unstimulated control cultures were 0.029, 0.031 and 0.027 in naïve, Rural 1 and Rural 2 subjects, respectively. (D – F) 6 day IL-10 responses in rural individuals with or without antibodies to (D) PfSE or (E) recombinant malaria antigens, or (F) individuals with or without detectable memory B cells specific for *P. falciparum* antigens. [Horizontal line  =  median; box  = 25^th^ and 75^th^ percentiles; whiskers  =  minimum and maximum values]. Mann-Whitney U test was used to analyse differences between groups.

Immediate (24 hr) IFN-γ responses to PfSE were significantly higher in individuals with serum antibodies to PfSE (Figure 4A) or with serum antibodies to at least one recombinant *P. falciparum* antigen (apical membrane antigen-1, merozoite surface protein (MSP)-1, MSP-2 and circumsporozoite protein) (Figure 4B) than in individuals who were seronegative for all of these antigens; individuals responding to three or more antigens had the highest IFN-γ responses. Furthermore, immediate (24 hr) IFN-γ responses to PfSE were also significantly higher among individuals who had memory B cells against at least one *P. falciparum* antigen (as determined by ELISPOT assay) than among those who had no detectable memory B cells (Figure 4C). Similarly, PfSE-specific 6 day IL-10 responses were also higher in seropositive than in seronegative individuals (Figure 4D, 4E) although IL-10 responses were not different between subjects with memory B cells and those without (Figure 4F).

## Discussion

The induction and maintenance of immunological memory to malaria has been a topic of debate for many years (reviewed in 2,22,23) but there are remarkably few studies that have attempted to examine memory responses over time. We have recently observed that B cell memory responses are stably maintained for at least 6 years in a rural Thai population living in an area where *P. falciparum* and *P. vivax* are endemic but where transmission is kept at extremely low levels [16]. In an extension of this study, we have now examined both the short-term (12 months of the study) and long-term (history of infection in the past 6 years) stability of the CD4^+^ T cell memory responses to malaria antigens. We find that although immediate Th-1 effector memory responses (24 hr IFN-γ secretion from CD45RO^+^ CD4^+^ T cells) decay with a half-life of approx. 3 years, central memory regulatory responses (6 day accumulation of IL-10 in culture supernatants) are stably maintained for at least 6 years after last-documented malaria infection.

In line with previous studies [13], [15], [19], we observed that CD4^+^ T cells from both malaria-naïve and malaria-exposed individuals proliferated extensively when co-cultured with PfSE. Our observation that, even in malaria naïve individuals, the vast majority of these proliferating cells exhibited a memory (CD45RO^+^) phenotype is consistent with previous studies indicating that these cells have been primed by exposure to commensal micro-organisms, pathogens and/or vaccine antigens carrying minimal T cell epitopes that cross-react with those of malaria proteins. Presumably the cells differentiate into memory cells as a result of microbial priming and subsequently proliferate when exposed to cross-reacting malaria antigens. Moreover, lymphoproliferative, IFN-γ and IL-10 responses to PfSE were so similar among cells from individuals known to have been previously infected with *P. vivax* and those known to have been infected with P. *falciparum* that we were able to pool the data for *P. vivax* and *P. falciparum*-infected subjects for all analyses. Pooling of these data was appropriate since there is extensive antigen cross-reactivity between crude *P. falciparum* and *P. vivax* extracts [24] and thus individuals who were seropositive for *P. falciparum* antigens may well have been exposed to *P. vivax,* and vice versa.

Despite the lack of specificity of the T cell proliferative responses, malaria antigen-induced effector functions – immediate (24 h) IFN-γ-producing effector memory cells and cultured (day 6) IL-10 responses - were seen only in malaria-exposed subjects. These observations suggest that – at least among malaria-naïve donors – many of the cross-reactive proliferating cells are relatively undifferentiated and may best be described as Th0 rather than Th1 or regulatory cells. Conversely, among subjects with known malaria infection in the past 6 years, a substantial proportion of these cells fit the phenotype of effector memory Th1 cells, suggesting either that malaria infection is required to drive Th1 differentiation of cross-reactive memory cells or that malaria infection induces Th1 differentiation of additional T cell populations (possibly of differing antigen specificity).

Interestingly, we found no evidence of immediate effector cells producing IL-10; IL-10 secretion seemed to occur significantly later (accumulating over 6 days) suggesting that regulatory responses may reside within the central memory population and that their reactivation may be secondary to activation of Th1 cells. Whilst the cellular source of IL-10 in 6 day culture supernatants is not known, it was secreted in an antigen-specific manner. B cells secreting IL-10 in an antigen-specific manner have been described in mice but the antigen specificity of IL-10-secreting B cells is poorly documented in humans and they are found at much lower frequencies than IL-10 producing T cells [25]. Although NK cells and monocyte/macrophages are potential sources of IL-10, their responses are not expected to be antigen specific or to differ between exposed and unexposed individuals. By analogy to studies in children recovering from acute malaria infections, one likely source of IL-10 is a population of CD4^+^ CD25^−^ Foxp3^−^ memory cells [26] but further studies are required to test this supposition. Both IFN-γ and IL-10 responses were more prevalent in subjects who had malaria-specific antibodies and/or memory B cells suggesting – unsurprisingly, perhaps - that the three arms of the adaptive immune response (cellular, humoral and regulatory) are induced in a coordinated fashion.

The significant decline in the magnitude of the IFN-γ effector memory response with time since last known malaria infection was in stark contrast to the highly stable nature of the humoral immune response and of the IL-10 regulatory response and suggests that IFN-γ-producing cell lineages are less stable than IL-10-producing lineages. In support of our observations, secreted IL-10 responses to malaria peptides were detected in adults in a malaria epidemic-prone area of Kenya after several years of low transmission [27], and were stably detected over an interval of 9 months in an area of high transmission [28], but IFN-γ ELISPOT responses were much less stable in both settings. The half life of serum antibodies, memory B cells [16] and 6 day IL-10 responses did not differ significantly from infinity in the very same individuals in whom IFN-γ effector responses declined with a half-life of ∼3 years. These differing decay rates indicate that even though different aspects of the immune response may be induced in a coordinated fashion, they have different requirements for long term maintenance. On the other hand, given its well-described role as a B cell growth and differentiation factor [29], [30] and its role in controlling production of malaria-specific IgG [31], [32], long term maintenance of memory B cells may well benefit from the sustained antigen-specific IL-10 responses.

The relatively short half-life of Th1 effector cells compared to the long term maintenance of IL-10-secreting cells might be expected to lead to a shift, with increasing time since last malaria infection, of the balance of the anti-malarial immune response from a pro-inflammatory response towards a more anti-inflammatory response. This expectation is not entirely consistent with clinical observations in migrants, in whom re-exposure to malaria after many years typically results in low parasite densities (implying retention of anti-parasitic effector mechanisms) accompanied by quite significant clinical discomfort (reviewed in 23). On the other hand, long term retention of anti-malarial antibodies [16] may account for the ability to contain parasitaemia and the very low levels of severe malarial disease in these patients may well be due to long-lived regulatory responses [23].

Effector IFN-γ responses to malaria antigens appear to be rather short-lived, at least as compared to those induced by vaccination against viral diseases. Data on the longevity of immediate IFN-γ-producing CD4^+^ T cell responses are, surprisingly, rather few since most studies have tended to use IFN-γ ELISPOTS to enumerate these cells and thus the precise identity of the IFN-γ-producing cells (CD4 T cell, CD8 T cell or even NK cell [33]) is frequently unknown. However, Combadiere *et al*. [34] found that 20% of smallpox vaccinees retained circulating immediate IFN-γ-producing cells more than 13 years after vaccination with no obvious decline in the response between 13 and 25 yrs post-vaccination; although these responses were assessed by ELISPOT the cytokine-producing cells were shown to be CD4^+^ T cells in a follow-on study [35]. Similarly, Hanna-Wakim *et al* [36] detected mumps virus-specific immediate IFN-γ^+^ CD4^+^ T cells in all 10 individuals who had received mumps vaccination at least 10 years previously. Conversely, we observed that none of the individuals exposed to malaria more than 4 years ago had detectable malaria-specific immediate IFN-γ^+^ CD4^+^ T cells. It is not clear whether this reflects a true difference in duration of immune memory to viruses and malaria or whether it simply reflects differences between natural infection and vaccination.

IFN-γ secreting effector T cells recognizing the *P. falciparum* thrombospondin-related adhesive protein (TRAP) have been reported to be short-lived, on the basis that IFN-γ ELISPOT responses to individual TRAP peptides were unstable from one year to the next [37]. However, it is possible that the precursor frequency of Th1 effector memory cells to individual malaria antigens hovers around the detection threshold for the assay and is thus subject to random fluctuation, as previously suggested [27], [38]. The apparent acquisition of IFN-γ responses in some members of this study cohort over the 12 month period of the study is consistent with this interpretation and similar observations were made for IFN-γ responses to fragments of malarial merozoite proteins [38]. On the other hand, 56% of the rural subjects who made an immediate T cell IFN-γ response to PfSE at the first cross-sectional bleed also responded at the survey 12 months later suggesting that effector cell numbers remain substantially above the limit of detection in these individuals. Of interest however, the half-life of IFN-γ responses to malaria antigens appeared substantially (although, with this sample size, not significantly) longer in individuals previously infected with *P. vivax* than in those previously infected only with *P. falciparum*. Using a similar study design to the one we have used here, Zevering et al also observed a discernable decline in IFN-γ responses to the *P. falciparum* circumsporozoite protein within two years of the most recent malaria infection [39] and again, responses were much more stable over time among individuals previously infected with *P. vivax* [40]. Taken together these data suggest that persistence of dormant hypnozoite stages in the livers of *P. vivax*-infected subjects may provide a source of persisting antigen for efficient renewal of effector memory cells.

Although there was a clear decline in the frequency of malaria antigen-specific IFN-γ secreting effector T cells with time since last known malaria infection, the half-life of these cells is still orders of magnitude greater than the lifespan of an individual effector memory cell. It has been estimated that effector memory and central memory T cells disappear from the peripheral circulation with half-lives of 6 days and 17 days respectively [41]. Thus, to persist for many months or years, memory T cells must either reside in tissues for extended periods of time or undergo relatively frequent self-renewal. Whilst we cannot formally exclude the possibility that effector cells are persisting – undetected - in tissues, we consider it unlikely. Only activated lymphocytes expressing adhesion molecules and chemokine receptors are able to migrate across endothelial barriers and gain access to non-lymphoid compartments and the endothelial barrier itself also needs to be activated (e.g. by inflammation) for this to occur. Whilst trafficking into the liver might occur in subjects with *P. vivax* hypnozoites, this would not be expected in subjects previously infected with *P. falciparum*. Cells remaining within the lymphoid compartment would be expected to recirculate and thus should appear, transiently but regularly, in the blood where they would be available for sampling. Conversely, intermitotic intervals of 15-50 days for memory T cells [41] are consistent with the self-renewal hypothesis and periodic exposure to persisting or cross-reacting antigens offers an obvious means by which this might happen.

In summary, our data demonstrate the acquisition of specific, anti-malarial effector memory IFN-γ and central memory IL-10 responses in individuals undergoing very infrequent exposure to malaria infection. Although larger studies are required to more accurately estimate the longevity of IFN-γ and IL-10 responses, the apparently very different half-lives of these two responses raise interesting questions regarding the requirements for survival and self-renewal of these two populations of cells. Although it remains possible that persistent and repeated malaria infections in areas of very high endemicity may eventually lead to T cell anergy or clonal exhaustion, the fact that individuals in these areas eventually become resistant to high density malaria infections and clinical symptoms argues against this as a major impediment to development of effective immune responses. Finally, if the ∼3 year half life for effector memory responses to PfSE is typical for malaria antigens, and if responses of similar durability can be induced by vaccination, our results could be seen as encouraging for vaccine developers since they imply that – once induced – anti-malarial immune responses are likely to persist for long enough to confer a reasonable degree of protection even in the absence of frequent boosting.

## Supporting Information

Figure S1 Immediate IFN-γ and day 6 IL-10 responses to PPD. (A) Fold increase (mean ±95% CI) in IFN-γ production in response to PPD (compared with cells cultured without antigen) at recruitment for each subject. (B,C) Immediate IFN-γ at recruitment compared to at 12 months later in Rural 1 (B) and Rural 2 (C) subjects. Horizontal line  =  median; box  = 25th and 75th percentiles; whiskers  =  minimum and maximum values. Paired t-tests were used to analyse differences between the two time points. (D) Concentration (mean ±95% CI) of IL-10 in 6 day, PPD-stimulated culture supernatants at recruitment. (E,F) IL-10 concentrations in 6 day, PPD-stimulated culture supernatants at recruitment and 12 months later in Rural 1 (E) and Rural 2 (F) subjects. Horizontal line  =  median; box  = 25th and 75th percentiles; whiskers  =  minimum and maximum values. Paired t-tests were used to analyse differences between the two time points.
(0.20 MB PPT)

Table S1 The mean ± SD of the percentages of CD45RO+ CD4+ T cells that divided (i.e. were CFSElow) after in vitro restimulation with PfSE, PPD or PHA.
(0.13 MB PPT)
