# Supplementary material for: Short-Lived IFN-γ Effector Responses, but Long-Lived IL-10 Memory Responses, to Malaria in an Area of Low Malaria Endemicity
Source: PLoS Pathog. 2011 Feb 10;7(2):e1001281. doi: 10.1371/journal.ppat.1001281 (PMC3037361; doi:10.1371/journal.ppat.1001281)
Supplement: Figure S1 — Immediate IFN-γ and day 6 IL-10 responses to PPD. (A) Fold increase (mean ±95% CI) in IFN-γ production in response to PPD (compared with cells cultured without antigen) at recruitment for each subject. (B,C) Immediate IFN-γ at recruitment compared to at 12 months later in Rural 1 (B) and Rural 2 (C) subjects. Horizontal line = median; box = 25th and 75th percentiles; whiskers = minimum and maximum values. Paired t-tests were used to analyse differences between the two time points. (D) Concentration (mean ±95% CI) of IL-10 in 6 day, PPD-stimulated culture supernatants at recruitment. (E,F) IL-10 concentrations in 6 day, PPD-stimulated culture supernatants at recruitment and 12 months later in Rural 1 (E) and Rural 2 (F) subjects. Horizontal line = median; box = 25th and 75th percentiles; whiskers = minimum and maximum values. Paired t-tests were used to analyse differences between the two time points. (0.20 MB PPT) [file ppat.1001281.s001.ppt]

## Slide 1
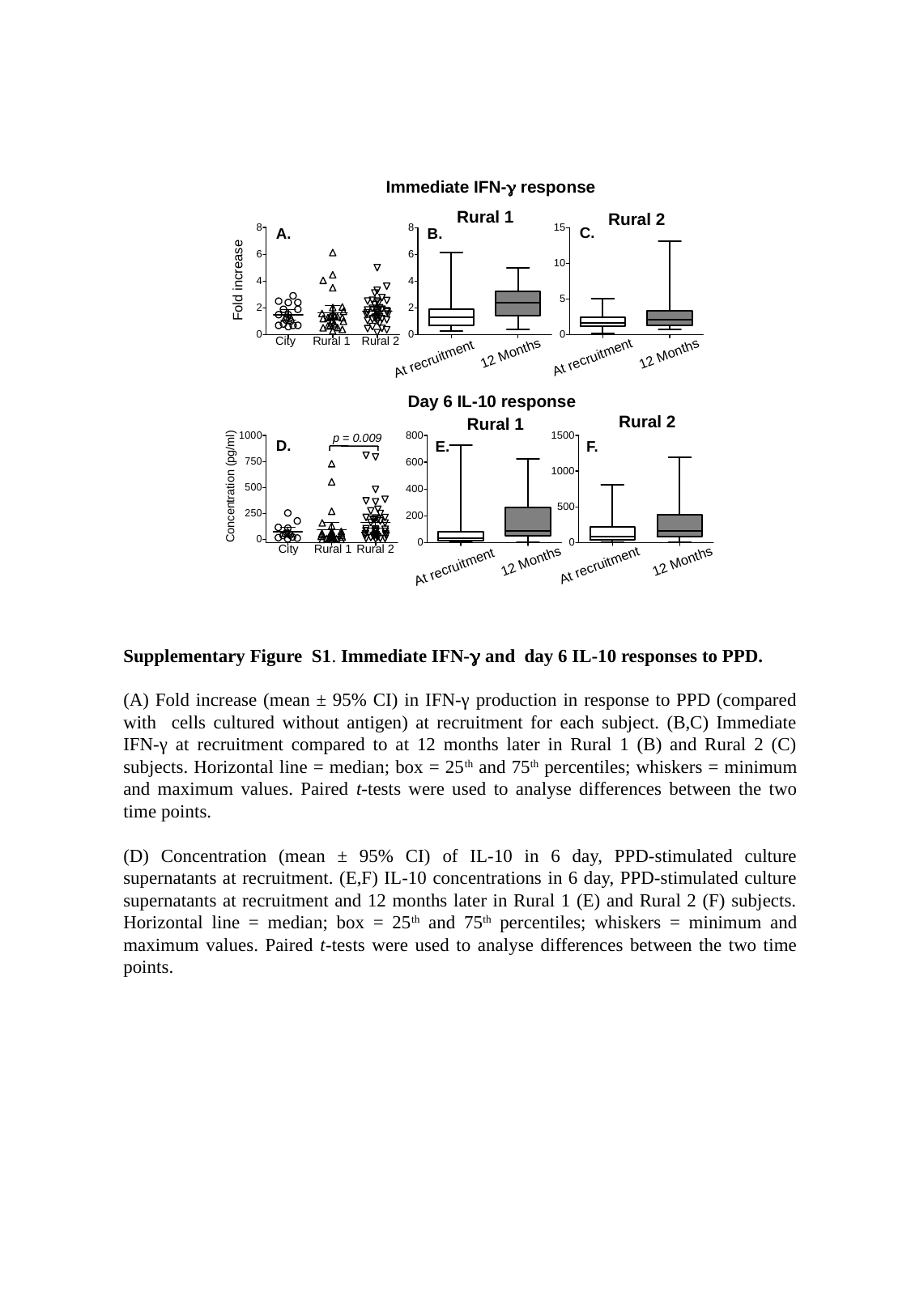

Immediate IFN-γ response
Rural 1
Rural 2
C.
A.
B.
Fold increase
City
Rural 1
Rural 2
12 Months
12 Months
At recruitment
At recruitment
Day 6 IL-10 response
Rural 2
Rural 1
p = 0.009
D.
E.
F.
Concentration (pg/ml)
City
Rural 1
Rural 2
12 Months
12 Months
At recruitment
At recruitment
Supplementary Figure S1. Immediate IFN-γ and day 6 IL-10 responses to PPD.
(A) Fold increase (mean ± 95% CI) in IFN-γ production in response to PPD (compared with cells cultured without antigen) at recruitment for each subject. (B,C) Immediate IFN-γ at recruitment compared to at 12 months later in Rural 1 (B) and Rural 2 (C) subjects. Horizontal line = median; box = 25th and 75th percentiles; whiskers = minimum and maximum values. Paired t-tests were used to analyse differences between the two time points.
(D) Concentration (mean ± 95% CI) of IL-10 in 6 day, PPD-stimulated culture supernatants at recruitment. (E,F) IL-10 concentrations in 6 day, PPD-stimulated culture supernatants at recruitment and 12 months later in Rural 1 (E) and Rural 2 (F) subjects. Horizontal line = median; box = 25th and 75th percentiles; whiskers = minimum and maximum values. Paired t-tests were used to analyse differences between the two time points.
