# Supplementary material for: Short-Lived IFN-γ Effector Responses, but Long-Lived IL-10 Memory Responses, to Malaria in an Area of Low Malaria Endemicity
Source: PLoS Pathog. 2011 Feb 10;7(2):e1001281. doi: 10.1371/journal.ppat.1001281 (PMC3037361; doi:10.1371/journal.ppat.1001281)
Supplement: Table S1 — The mean ± SD of the percentages of CD45RO+ CD4+ T cells that divided (i.e. were CFSElow) after in vitro restimulation with PfSE, PPD or PHA. (0.13 MB PPT) [file ppat.1001281.s002.ppt]

## Slide 1
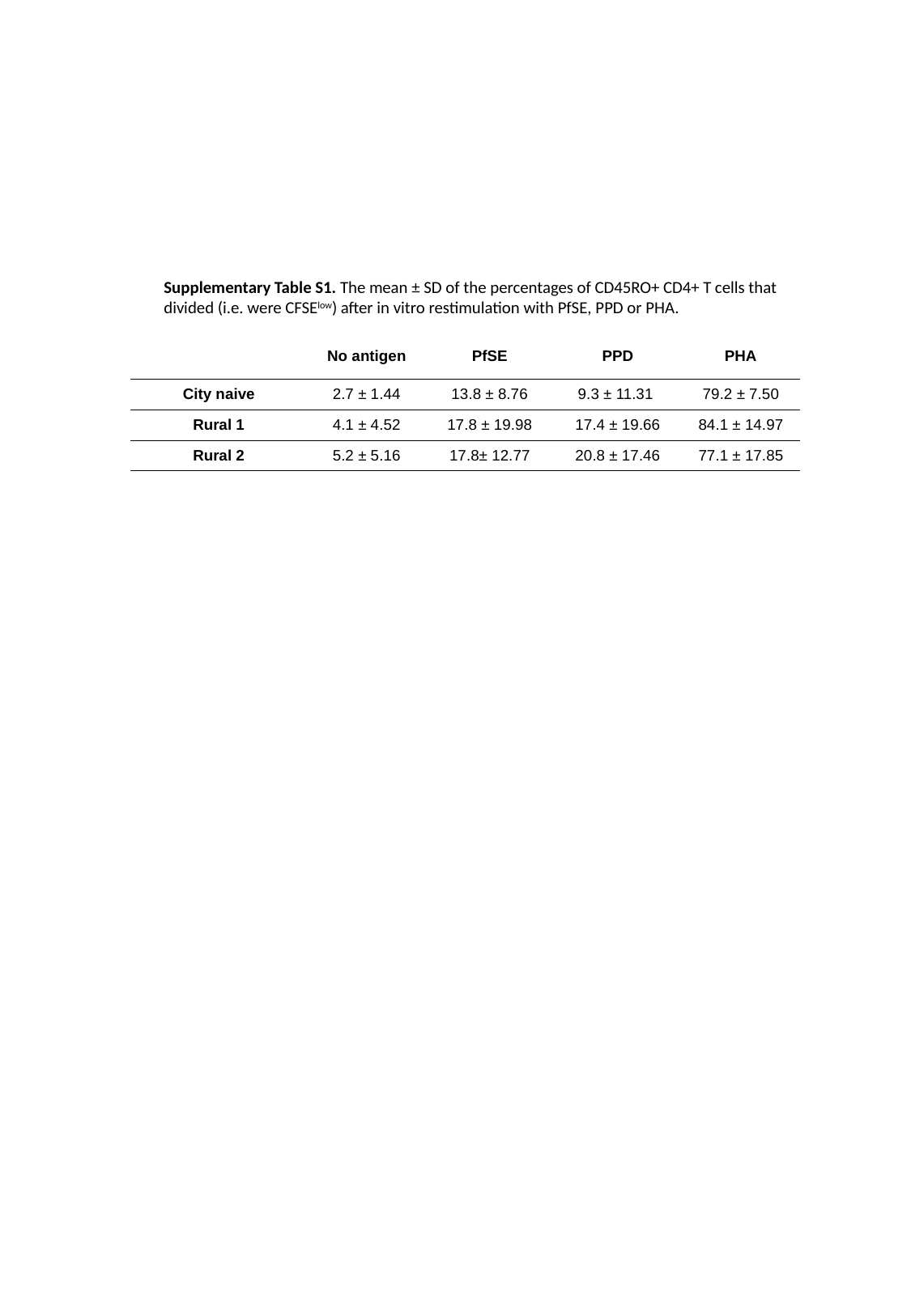

Supplementary Table S1. The mean ± SD of the percentages of CD45RO+ CD4+ T cells that divided (i.e. were CFSElow) after in vitro restimulation with PfSE, PPD or PHA.
| | No antigen | PfSE | PPD | PHA |
| --- | --- | --- | --- | --- |
| City naive | 2.7 ± 1.44 | 13.8 ± 8.76 | 9.3 ± 11.31 | 79.2 ± 7.50 |
| Rural 1 | 4.1 ± 4.52 | 17.8 ± 19.98 | 17.4 ± 19.66 | 84.1 ± 14.97 |
| Rural 2 | 5.2 ± 5.16 | 17.8± 12.77 | 20.8 ± 17.46 | 77.1 ± 17.85 |
